# Mycosis fungoides bullosa: a case report and review of the literature

**DOI:** 10.1186/1752-1947-4-78

**Published:** 2010-03-03

**Authors:** Hermann Kneitz, Eva-B Bröcker, Jürgen C Becker

**Affiliations:** 1 Department of Dermatology, Julius-Maximilians University, Josef-Schneider-Str. 2, 97080 Wuerzburg, Germany

## Abstract

**Introduction:**

Mycosis fungoides, the most common type of cutaneous T-cell lymphoma, can manifest in a variety of clinical and histological forms. Bulla formation is an uncommon finding in mycosis fungoides and only approximately 20 cases have been reported in the literature.

**Case presentation:**

We present a case of rapidly progressive mycosis fungoides in a 68-year-old Caucasian man who initially presented with erythematous plaques characterised by blister formation.

**Conclusion:**

Although mycosis fungoides bullosa is extremely rare, it has to be regarded as an important clinical subtype of cutaneous T-cell lymphoma. Mycosis fungoides bullosa represents a particularly aggressive form of mycosis fungoides and is associated with a poor prognosis. The rapid disease progression in our patient confirms bulla formation as an adverse prognostic sign in cutaneous T-cell lymphoma.

## Introduction

Mycosis fungoides, the most common type of cutaneous T-cell lymphoma, can manifest in a variety of clinical and histological forms, but blistering is not a feature normally associated with the condition. Indeed, of the many variants that have been reported in the literature, approximately 20 cases of the bullous variant have been described.

## Case presentation

**Figure 1:**
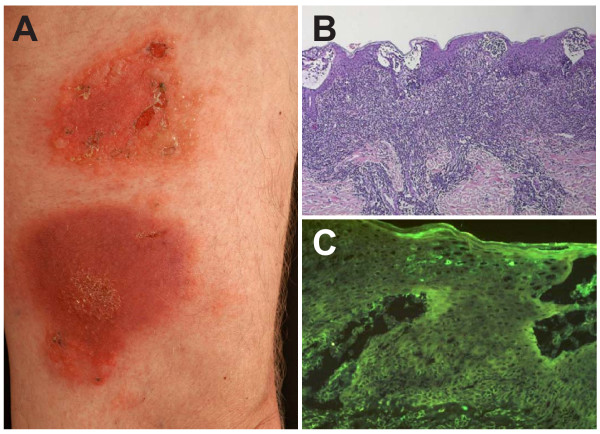
**Mycosis fungoides presentation, and histological and immunohistochemical examinations**. (a) Bullous lesions are present on two indurated erythematous plaques on the left thigh. (b) Histopathologic section showing subcorneal and intra-epidermal bullae accompanied by infiltrates of atypical lymphocytes. (c) Direct immunofluorescence examination reveals the absence of immunoreactants both within the epidermis and at the dermo-epidermal junction.

A 68-year-old Caucasian man presented for evaluation of two indurated erythematous plaques (9 × 8 cm and 8 × 5 cm) on his left thigh (Figure 1A); within these plaques, intact bullae and erosions were present. The condition had persisted for several years with a slow growth in diameter and thickness despite topical therapy with potent steroids. Notably, the patient reported several fugacious episodes of blistering within and in the vicinity of these plaques; these episodes had occurred with increasing frequency over the preceding months. The general examination results were otherwise unremarkable. In particular, neither lymphadenopathy nor organomegaly were present. Histological examination of these lesions revealed subcorneal and intra-epidermal bullae accompanied by infiltrates of atypical lymphocytes. The latter were characterised by a marked epidermotropism and the formation of Pautrier microabscesses (Figure 1B). Immunohistochemical analysis revealed the infiltrate to be predominantly T cells (CD3+, CD20-). Direct (Figure 1C) and indirect immunofluorescence as well as bacterial and fungal cultures were negative.

Initial treatment of our patient with electron beam irradiation led to complete clearance, but after 1 year, generalised erythematous plaques of mycosis fungoides bullosa appeared on his trunk and extremities without lymph node and visceral involvement. Further electron beam irradiation was carried out.

## Discussion

The most common causes of acquired bulla formation on inflamed skin areas are acute contact dermatitis as well as infections with *Staphylococci*, or viruses of the herpes group. Bullous lichen planus or bullous lupus erythematosus are rare diseases, whereas bullous pemphigoid is the most common autoimmune bullous disorder [1].

In mycosis fungoides, bulla formation is very uncommon. Approximately 20 cases have been reported in the literature [2,3]. Mycosis fungoides bullosa is largely restricted to older patients without predominance of gender. Predilection sites are the trunk and limbs. Vesicles and blisters usually arise in typical plaques and tumours but also in normal-appearing skin [2,4].

An association with concomitant bullous pemphigoid or previous treatment with psoralen UVA photochemotherapy has been reported [5]. Bowman *et al*. proposed the following criteria for diagnosis of mycosis fungoides bullosa [2]: (1) clinically apparent vesiculobullous lesions; (2) typical histologic features of mycosis fungoides (atypical lymphoid cells, epidermotropism, Pautrier's microabscesses) with intra-epidermal or subepidermal blisters; (3) negative immunofluorescence ruling out concomitant autoimmune bullous diseases; and (4) negative evaluation for other possible causes of vesiculobullous lesions (for example, medications, bacterial or viral infection, porphyria, phototherapy).

The pathological mechanism underlying blister formation has not been elucidated. Confluence of Pautrier's microabscesses in mycosis fungoides lesions may lead to intra-epidermal bulla formation [6]. Alternatively, proliferation of neoplastic lymphocytes may result in a loss of coherence between basal keratinocytes and basal lamina [7] or the cohesion of keratinocytes may be affected by the release of lymphokines by atypical lymphocytes [8].

## Conclusions

Although mycosis fungoides bullosa is extremely rare, it has to be regarded as an important clinical subtype of cutaneous T-cell lymphoma. Mycosis fungoides bullosa represents an especially aggressive form of mycosis fungoides associated with a poor prognosis. Approximately 50% of patients die within 1 year after the appearance of the blistering of the lymphoma plaques [1,9].

## Consent

Written informed consent was obtained from the patient for publication of this case report and any accompanying images. A copy of the written consent is available for review by the Editor-in-Chief of this journal.

## Competing interests

The authors declare that they have no competing interests.

## Authors' contributions

All authors contributed in the management of the patient, writing of the manuscript and reviewing the literature. All authors read and approved the final manuscript.
